# Improving collaborative care networks for functional disorders and persistent somatic symptoms: a participatory action research study in the Netherlands

**DOI:** 10.1136/bmjopen-2025-107978

**Published:** 2025-12-12

**Authors:** Nick Mamo, Denise J C Hanssen, Sylvia Korten, Tim C Olde Hartman, Judith GM Rosmalen, Lineke Tak

**Affiliations:** 1 Department of Psychiatry, University Medical Centre Groningen, Groningen, The Netherlands; 2 Alkura Specialist Center Persistent Somatic Symptoms, Dimence Groep, Deventer, The Netherlands; 3 ALK Netwerk Salland, Deventer, The Netherlands; 4 Department of Primary and Community Care, Radboud universitair medisch centrum, Nijmegen, The Netherlands; 5 Department of Internal Medicine, UMCG, Groningen, The Netherlands

**Keywords:** Community-Based Participatory Research, Implementation Science, PSYCHIATRY, Patient Care Management, Delivery of Health Care, Integrated, Chronic Disease

## Abstract

**Abstract:**

**Background:**

Persistent somatic symptoms and functional disorders are conditions requiring a biopsychosocial approach to care, often from multiple professionals. The fragmentation of care common in most health systems results in unsatisfactory and challenging care experiences. Collaborative care networks form an important route towards improving outcomes and the overall experience of care for patients and professionals. While we have a good idea of what such collaborative care networks can look like, we lack knowledge on the practicalities of implementing change in such networks.

**Objectives:**

The core objective of this study is to implement change in a collaborative care network for persistent somatic symptoms and functional disorders care. Our questions were twofold: first, what are examples of realistic action processes to improve such collaborative care networks? Second, what are, in our experience, conditions for an effective change process in such a collaborative care network?

**Design:**

Participatory action research approach embedded within an active regional network between May 2023 and May 2024. The process was led by an action group who selected objectives and related actions with the aim of improving the network, leading to better care for people with persistent somatic symptoms and functional disorders as well as improving satisfaction among professionals.

**Setting:**

*ALK Netwerk Salland,* a regional network of professionals and experts-by-experience, focused on care of persistent somatic symptoms. This network is based in the *Salland* region in the east of the Netherlands, centred around the city of Deventer.

**Participants:**

The action group was made up of local stakeholders including experts-by-experience and health and social care professionals, facilitated by a researcher-in-residence. Other participants included members of the regional network who provided input towards the different objectives.

**Results:**

Over the course of a year, three objectives were selected and enacted, including assessing the resources of the network, improving knowledge of treatment options and improving the shared vision of care. The process faced some challenges, such as changes in action group members and a lack of resources and time to enact changes. However, by having a trusted and engaged team, working with an active network, we were able to enact significant changes to the network, which may be sustained and built on through the ongoing action group.

**Conclusions:**

Future participatory action research studies would benefit from a trusted and embedded researcher-in-residence, meaningful involvement early in the process of experts-by-experience, and serious consideration of realistic outcome measures to monitor for evaluation of changes made.

**STRENGTHS AND LIMITATIONS OF THIS STUDY**

- Measuring outcomes was often not easy or even possible. Often, the products themselves had to be taken as the outcome rather than measuring the product’s impact.
- The work of the action group directly reflects the experiences and needs of the members of the action group and the wider network.
- Having a diverse action group, and especially the involvement of experts-by-experience, allowed for triangulation of ideas and strong critique, to strengthen plans.
- An important strength inherent in the participatory action research method is the ability to use research methods and develop actions based on the needs and resources of the network in the current time.
- The study was designed in a way to be sustainable and continue once the study time ended. This resulted in a more motivated action group, a focus on the work not the research, and a framework within which the action group and network could move forward.

## Introduction

Persistent somatic symptoms (PSS) are distressing physical symptoms lasting for several months, irrespective of their cause.^1^ PSS occur in the context of a wide range of somatic diseases, mental disorders and functional disorders (FD), where they present in recognised clusters of symptoms.^1^ Often also referred to as functional somatic disorders, FDs include multiple disorders such as functional neurological disorder and irritable bowel syndrome, among others. Such disorders are typified by recognised clusters of PSS. Both PSS and FD are influenced by multiple biopsychosocial factors, which may affect as predisposing, precipitating and/or perpetuating factors.^2^ For this reason, treating PSS/FD requires a biopsychosocial approach to care often from multiple professionals from different disciplines.^3^ Unfortunately, current care systems are fragmented with a lack of integration within and between healthcare services and a lack of collaboration between care professionals.^4 5^ This lack of collaboration impacts both those suffering from PSS/FD and the professionals themselves, with both groups having poor experiences.^6 7^ The health outcomes of those suffering from PSS/FD are also negatively affected, with delays to diagnosis and treatment.^8^

Interprofessional collaboration is seen as an important route to improve outcomes for persons with PSS/FD as well as the overall care experience for patients and professionals.^9^ This collaboration can be improved through the development of collaborative care networks (CCNs), in which professionals work together to provide patient-centred care for PSS/FD.^10^ A previous Delphi study among professionals involved in PSS/FD care identified various quality indicators for such CCNs, including open communication, a shared vision of care and pathways tailored to the individual patient ^11^. At face value, some of these quality indicators may seem easy to adopt. However, there is a difference between identifying solutions to improve CCN quality and applying them in daily practice. Implementation can be a long, complex process facing many barriers on the way and must be specific to the context where the CCN is (to be) set up.^12^ Some barriers are more general, such as a lack of guidelines or management support.^13^ Other barriers would be more specific to PSS/FD care, such as stigma,^14^ or are related to collaboration, such as lack of a shared language.^15^ Barriers may also be related to the local or national context, such as national financial structures not being flexible enough for the specific adaptations needed^16^ as well as access-related issues or limited resources.^9^ These barriers would require tailored solutions and implementation strategies, for example, use of the same unambiguous terminology across levels of care,^15^ or creating a network map showing relevant care providers.^17^ Waiting until national financial healthcare structures are settled or funding is granted to implement change projects is not realistic, partly because of the paradoxical truth that often these only follow once certain strategies have been developed and proven themselves in practice through a bottom-up approach.

The next step, therefore, is implementing changes in practice. Very little work has focused on the process of implementing change in CCNs for PSS/FD care. Our questions were twofold: first, what are examples of realistic action processes to improve CCNs for PSS/FD care? Second, what are, in our experience, conditions for an effective change process in a CCN for PSS/FD?

To answer these questions, we took a participatory action research (PAR) approach. The PAR approach is one based on core principles of participation of relevant stakeholders and embeddedness within the communities most affected by the services involved and the changes to them, rather than a set research design.^18 19^ Different research methods are employed within this approach depending on the objectives and actions decided on by the group within the community entrusted with deciding on the action processes to take. PAR is, therefore, a research approach that would provide realistic action processes, created by the persons who form, and work within, the network.

## Methods

The current study is part of the innovative training network Encompassing Training in fUnctional Disorders across Europe (ETUDE), ultimately aiming to improve the understanding of mechanisms, diagnosis, treatment and stigmatisation of FD.^4^

### Study design

**Figure 1:**
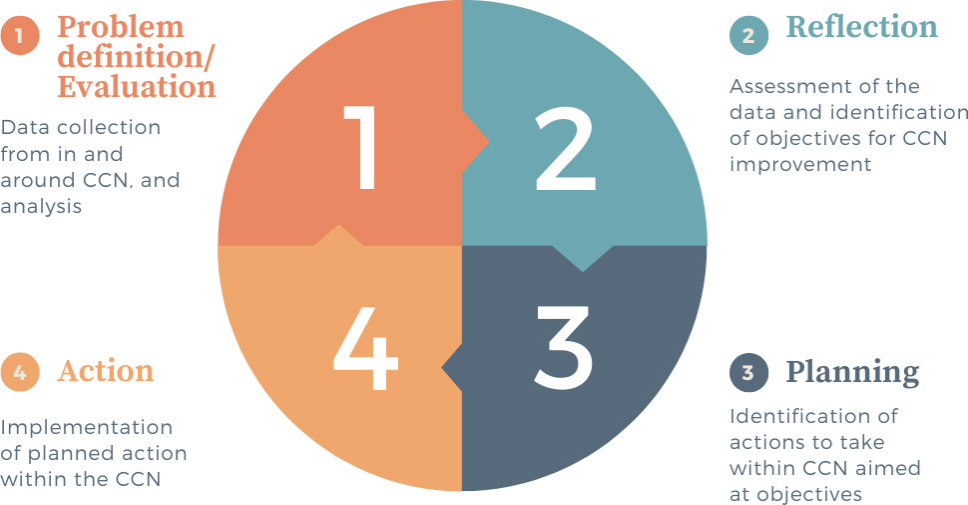
Participatory action research cycle. CCN, collaborative care network.

As a means to identify effective action processes for improving an active CCN in PSS/FD care, this study utilised a PAR approach. The core research process of PAR is primarily based on a cyclical process of action and critical reflection.^20^ We have presented it as: (1) problem definition—including data collection and analysis, evaluation of outcomes of previous completed cycles if applicable; (2) reflection on the outcomes of the analysed data and evaluation, with identification of change objectives; (3) planning of actions targeting the change objectives reflecting the analysed data and (4) action—implementing planned actions (see figure 1). This study design differs from quality improvement projects, in particular, those using the plan-do-study-act cycle often seen in healthcare. The primary difference lies in PAR’s focus on active participation of key stakeholders who form part of the healthcare service. In particular, this includes the ‘service users’—persons with PSS/FD who have needed or currently need the healthcare service. Where this study differs from other PAR studies used in healthcare improvement^21^ is in the fact that data specifically relevant for improving CCNs for PSS/FD were already available.^1011 14^,^16^ Other data sources were also considered and consulted, such as a questionnaire and ideas on strategies for improving quality developed from quality indicators identified in a Delphi study.^11^ As the action group decided to use some of the data available, the process could be more efficient, by shortening the first step of the PAR cycle, moved forward by the data available onto the reflection and planning stages.

Sustainability was another important principle in the process, which was designed in such a way that the action group could continue their work after this study was due to conclude.

### Study setting

The study was undertaken in the Netherlands and the Dutch healthcare system. This is a system based on an insurance model, with primary care acting as gatekeepers to secondary care access.^22^ While there is a growing interest in collaborative and integrated care, its availability remains limited, with challenges related especially to funding and coordinating care, with no funding for multidisciplinary consultations.^17^ It is common to find solo practitioners from a variety of disciplines working in the community, providing generally a primary care service, though often with special areas of interest. There are specialised PSS/FD services available in some provinces, though in most cases, management is through family doctors, or through specialties related to the main symptoms.

Specifically, this study took place within *aanhoudende lichamelijke klachten* (*ALK) Netwerk Salland*, which is a voluntary, regional network made up of health and social care professionals providing care for persons with PSS/FD as well as experts-by-experience. The network aims to improve the connections between healthcare and social care practitioners providing personalised integrated care for people with PSS/FD (‘*ALK*’ in Dutch). The network seeks to break down barriers between different departments and levels of care alongside working more closely with experts-by-experience (persons with lived experience of PSS/FD). This network is run by a steering committee who all volunteer in their roles, and as a network has a general meeting two times a year. The general meetings have a theme (ie, ‘children and PSS’ or ‘chronic pain’), with presentations related to the theme and pitches given by different professionals working in the region. There is also space for interactive workshops and member networking. During the course of the study, a network coordinator was employed for a trial of 1 year, on a part-time basis. Their duties comprise of improving governance in the networks as well administration of the network. Administration includes managing the mailing list and website as well as coordinating and supporting in the planning of upcoming meetings.

### Participants

The people involved in a PAR study depend on the relevant stakeholders in the community or network involved.^23^ The primary people involved in this case formed the action group—defining the data to be collected, the methods of analysis and the actions to be taken in response. The action group was made up of *ALK Netwerk Salland* members, each representing one or more of the six levels of healthcare as described by Grol and Wensing.^24^ The six levels are innovation, individual professional, patient, social context, organisational context and economic and political context. In this instance, we aimed for a group of approximately six to eight, as this allows for sufficient sharing of ideas without having the challenges of facilitating meetings with a large group. We aimed for a minimum of one person representing each level (though one person may represent two levels), and two persons representing the expert-by-experience level (patients/persons with lived experience of PSS/FD). The persons approached to be part of the action group were asked to commit to regular meetings for the duration of the study of 1 year. The role of the action group was to decide on objectives and actions to improve the active network using data already available as well as with the option of collecting and analysing new data. The role of the main researcher (NM) within this group was as process facilitator as well as being ‘researcher-in-residence’ (RIR—embedded within the organisation, working as a researcher and research translator).^21 25^ In this case, the role of RIR included organising and facilitating meetings and providing support through provision of relevant resources (including literature), enacting any research or data and support with enacting the plans of the action group. These factors assisted in ensuring the work done was participatory, embedded in the local context, as well as being reflexive and transparent. Reflexivity is the process of critical analysis on the part of the embedded researcher. This includes assessing power balance, influence on the research and action processes as well as other ethical questions.^26^

### Procedure and reporting

The study took place over a duration of 1 year from May 2023 until May 2024 with seven planned action group meetings. Meetings were held in hybrid format and were of maximum 2 hours duration. Meetings had an assigned chairperson and a preshared agenda. In general, the agenda introduced new members, reviewed ongoing actions and discussed future action, including any discussions needed with the steering committee or other network members.

During the first meeting, there was also a presentation of the study process and an explanation of the roles of different persons in the study. This included the proposal to use an objectives template developed for this study as a way to track the work of the action group. Alongside this, relevant previous study results concerning CCNs for PSS/FD were presented, which were proposed as a basis for the selection of objectives for the work of the action group. The quality indicators identified through a Delphi study were used as a basis for selecting objectives.^11^ The developed objectives template brought together the objectives defined by the action group, and their associated action plans, evaluation plans and outcomes. The completed versions of this provided our primary data (see template: osf.io/thm7a and completed forms: osf.io/fcwt5, osf.io/5brkh and osf.io/32zu9).

Analysis was dependent on the outcomes of the objectives. Further data were collected through field notes taken by NM throughout the process, as well as feedback from the action group on the study and the process (informally, through meetings and through a basic feedback form sent around the time of the final meeting). Data reporting takes into account reporting guidelines for PAR studies.^21 27^ This study was preregistered on the Open Science Forum: see online supplemental material or https://osf.io/983uz.^28^

### Patient and public involvement

Patient and public involvement is a core component of this study. While patients and public were not involved in the design of the study, their involvement was built into these research plans. The core element of this PAR study was to hand over control (including the decision-making for actions of the group) to a broad mix of network stakeholders. This included health and social care professionals, and, in particular, experts-by-experience, with a focus on ensuring that their voice is central to the action group’s work. The core research questions—identifying realistic action processes for improvement of CCNs and conditions for an effective change process in a CCN—were not defined with patient and public involvement. However, the actions of the action group and associated outcome measure were entirely in their hands, with as little involvement as possible in this process from the research team. Indeed, a lot of the work of the research team was focused on selecting and inviting an appropriate mixture of people to the action group, and ensuring their active engagement in the process. The RIR, while part of the action group, did not bring their own objectives to the group, but aimed to bring out the ideas from the other members, and encouraged actions that would bring in further patient and public involvement. Finally, another important aspect of this study is the aim of the action group being sustainable and continuing beyond the study period, with the RIR being replaced by another network member, and leaving the action group entirely in the hands of the network itself. This includes future dissemination work within the network and more widely, increasing the relevance to patients and public of the message being shared.

## Results

### Action group members

Aside from three of the study authors (NM, SK and LT), eight others were part of the action group at different times. This group included a family doctor, a physiotherapist, a project coordinator working in the social domain, a neurologist, a psychiatrist specialised in PSS/FD care, a psychologist and three experts-by-experience. NM was present in the group as the initiator of the PAR, and taking on the role throughout of RIR and the secretarial role, as well as chairing the first four meetings. Other network members also provided assistance from outside the action group.

The potential members of the action group were selected in discussion by NM and LT focusing on having a broad mix of stakeholders, representing the six levels of healthcare. From a professionals’ perspective, we aimed to include a mix from different disciplines, across both primary and secondary care as well as solo practitioners. The members of the action group were not specifically paid for their time in the study, either by their employers or from the study itself. For some, it was possible to use paid time to contribute, but this was not the case for all, in particular, the experts-by-experience.

Throughout the process, there were some changes in personnel. One expert-by-experience left shortly before the first meeting for health reasons, with another expert-by-experience replacing them in the action group. Another expert-by-experience left later in the process due to time and financial limitations. A psychologist joined the action group late in the process, as did the new network coordinator. Initially, LT was not directly involved in the action group, to avoid reliance on them as a recognised network leader. However, due to challenges with moving actions forward, following discussion between NM and LT, and with agreement from the action group, LT joined from the fourth session.

### Objectives and action processes

#### Process of objective selection

During the first meeting, the action group agreed to use the objectives form and to base the objectives on the quality indicators for CCNs in PSS/FD care identified in an earlier Delphi study.^11^ With this, the action group decided, as a first step, to ask the network meeting participants to recommend which indicators to focus on. This was done at the following network meeting, shortly after the first action group meeting, by asking ‘which three quality indicators do you think the action group should focus on’, providing the top 15 indicators identified in the Delphi study. The three highest-selected quality indicators by the participants at the network meeting were ‘awareness of the expertise of other disciplines’, ‘shared vision of care for PSS/FD’ and ‘clear overview of treatment options’.

With regards to the PAR cycle, only one cycle was mostly completed during the time included in this study, although an initial evaluation and reflection towards future plans were made. The actions from objective two were largely but not entirely complete, and some actions from objective three are ongoing by design. Discussion about next objectives began during the final meeting included in this study.

#### Selected objectives and associated action plans

Based on the discussions in the first meeting and the results of the network meeting questionnaire, three objectives were agreed on and actions taken (see table 1 and completed forms here for detailed action plans: osf.io/fcwt5, osf.io/5brkh and osf.io/32zu9). The selected objectives differed from the three highest-selected by the network meeting participants. This was due to an agreement within the action group that having other resources available would be useful in achieving objectives as well as recognising that ‘awareness of the expertise of other disciplines’ and ‘clear overview of treatment options’ overlap, and so, were combined.

**Table 1:** Objectives and action processes agreed on by action group

Objective		Actions		Evaluation	Outcomes
1	Assess network resources	1.1	Explore the possible role and involvement of social domain organisation, as well as resources and finances that may be accessed	Review of outcomes at following meeting	At present no resources directly available, but would need reconsidering in future
2	Improve knowledge of treatment options	2.1	Survey action group—understanding of PSS/FD, treatments offered and colleagues’ interest/understanding	Review responses from action group—any follow-up actions?	Knowledge of action was discussed within meetings, but survey was not completed online
		2.2	Review of feedback on PSS/FD resources, in particular websites	Review responses from action group—can these improve accessibility to the network?	Discussions during meeting. Helped form actions 2.3, 2.4
		2.3	Create overview document reviewing all relevant disciplines, add content to website	Document created? Available on website?	Document created with input from multiple disciplines. Content published on website
		2.4	Create videos to go alongside disciplines content in 2.3	Videos created? Available on website?	Most videos created, uploaded on online video-sharing platform for embedding onto website
		2.5	Promotion of produced content	Content promoted?	To be promoted at next network meeting, national PSS association meeting, online platforms
3	Improve shared vision of care for PSS/FD	3.1	Small-group interactive workshops during network meetings exploring elements of communication	Are the small-group workshops happening? Any feedback on these?	Two interactive workshops happened so far. Feedback has been positive with some learning. Second workshop had less feedback. To be taken into consideration for question to focus on for the following network meeting workshop
		3.2	Network and its work will be shared with family medicine trainees during a training event	Any feedback on presentation of network and its work? Expected impact on practice?	Training event not yet taken place at time of writing. Results will give an indication of usefulness

FD, functional disorder; PSS, persistent somatic symptoms.

With regards to the enacted three objectives, the main actions were:

1. *Assess resources of the network*
  Here, the action group wanted to identify any financial support we could access, which was unfortunately found to be restricted currently. This limits the actions we could take.
2. *Improve knowledge of treatment options*
  In this objective, the main idea was to create a clear overview of the treatment options and who provides the different treatment options, also improving awareness of the expertise of other disciplines.
  As an initial step, the action group for an overview of the knowledge and understanding within the group of what PSS and FD are, explanatory models and resources available.
  Following this, content was created including text and videos. The text was reviewed by professionals working in each of these disciplines and also reviewed by the experts-by-experience in the group, who also asked other experts-by-experience in their network to ensure the language is accessible to the general public (see the full list of disciplines for objective 2 here: osf.io/5brkh). This content has been published on the network website (www.alknetwerksalland.nl/behandelingen) with plans for videos for each discipline alongside. In particular, an introductory video by an expert-by-experience giving an overview will also be produced—because it was thought that seeing all information and videos at once may be overwhelming for a new patient. Plans were also made for the website to be improved with a more interactive interface.
3. *Improve shared vision of care for PSS/FD*
  Here, it was recognised that reaching a shared vision of care is likely to be a long process. However, by creating space where network members can talk about how they provide care and communicate with patients, a shared language and understanding can be created. This should preferably be alongside experts-by-experience sharing their experiences and how they think care should be provided and communication should take place. This would, therefore, also support a patient-centred view, and create a language that is realistic and relevant to patients and their care needs. With this in mind, the main action taken was to include interactive workshops during the 6 monthly network meetings where, in small groups, participants can role-play and discuss different elements of the treatment and communication process. In the first two interactive workshops (November 2023 and June 2024), the main questions for the groups were ‘what explanations do you provide to patients about what they are experiencing as PSS/FD?’ and ‘how do you involve patients in decision-making when planning treatment?’. At the end of each of these meetings, participants were asked (using an online questionnaire—see osf.io/qtvx9 and osf.io/85yuv for results) whether they learnt any sentences or models that they could use in their practice.

A possible fourth and fifth objectives were discussed in the last meeting in May 2024. The action group was primarily considering ‘involvement of the social domain’ and ‘active collaboration with somatic specialists’.

### Change process

A number of factors are recognised as important in the change process. Table 2 provided an overview of the most important ones.

**Table 2:** Factors impacting on the change process for the action groups

1	Action group	A	Part of network
		B	Representing relevant stakeholders—especially experts-by-experience
		C	Have understanding of the challenges
		D	Empowered and trusted by the CCN and its leadership
		E	Clear mandate from their organisation for work
		F	Room for informal connections to form
2	RIR/lead facilitator	a	Insider/outsider
		b	Team management and organisational skills
		c	Research skills
		d	Network connections
		e	Communication skills—in person and online
		f	Empowered and trusted by the CCN and its leadership
3	Resources	a	Financial support
		b	Time—for the process of change, for meetings and for trust to build within and beyond the action group
		c	Motivated network members
		d	Technical—including for hybrid meetings
		e	Continued support for further work beyond the specified time window for the project

CCN, collaborative care network; RIR, researcher-in-residence.

Where possible, informal meetings were held between the RIR and action group members before action group meetings began, or before the new member joined the action group in cases of the process having already begun.

Most action group members attended meetings in person, with one or two members joining online. Meeting in person was considered better for team-building and discussion quality, while agreeing that online attendance provided a way for attendance when physical presence was not possible. Absences were an issue due to scheduling difficulties related to work and childcare issues, with challenges in finding times agreeable to everyone due to different work days. Online attendance was in most cases due to the same reasons. Updates were provided by email after each meeting to ensure all members of the action group were up to date.

The first four meetings were chaired by NM. The following two meetings were chaired by different members of the action group. The last meeting included in this study was chaired by the new network coordinator as a transition step, with the aim of them chairing future meetings as the action group continues its work. This indeed took place, with further meetings planned and taking place by the time of writing, and new objectives being acted on.

The primary task for the action group was to agree on the objectives to focus on in their work. From this point, and for the rest of the first three meetings, the main discussion was about what the action group should prioritise. Aside from quickly agreeing with the suggestion of taking on two objectives, one relatively easily achievable and another requiring more time, decision-making on the objectives to take on was slow, and more so the actions to take for each. Meetings were very lively, with a lot of deep discussion, open disagreement and building consensus with both practical and conceptual discussion. A lot of discussion focused around what the network looks like, what matters to the network and therefore what should be dealt with first and would likely have an impact. This included a long discussion about the relevance to the network of the quality indicators from which we had agreed to pick our objectives. For this reason, meetings were longer than expected: 2 hours instead of the initial idea of 90 min. This is reflected by feedback after the last included meeting, with a reflection that a further introductory meeting to understand more what the action group was for would have been helpful and that ‘In the first meeting, we went pretty quickly into the agenda items to be achieved’. (action group member). One member of the action group fed back that they would have preferred more time to get to know each other, in particular, each other’s experiences and views, to improve social cohesion within the group. On the other hand, another group member felt that ‘There was just too much ‘talking around’ during the action group meetings without achieving anything’ *(feedback form response*). The process changed once action plans were decided on. From this point, the discussions were more focused, shifting to a task-driven approach.

Between meetings, the flow of communication was generally one sided. NM would send an update following meetings, including main outcomes and questions. However, in contrast to the meetings, engagement by email was very limited, often requiring specific reminders for certain questions to get responses. This was also reflected in receiving content from action group members and other network members related to specific actions. For most in the network, there is a lack of time for such activities, in spite of the clear motivation for the work of the action group. This was compounded by the lack of resources available, shown in the loss of a highly engaged action group member due to not having sufficient time and financial support. It was also reflected in the email replies from action group members as well as other network members who were asked for input. Often, persons would state that time to complete actions or produce content was only available many weeks in the future from the email being sent. Repeatedly, emails would then be received apologising for not sending replies and that clinical duties got in the way. This was also seen in some meetings where one member in particular would often join online from their clinic room a few minutes late due to clinic running over. As a result of the limited communication between meetings, all the decisions were made by those present at the meetings. The actions were, therefore, completed over a longer duration, with a lot of the content production being undertaken by LT. This related in particular to the production of the text for the website (action 2.3) and recruitment for videos (action 2.4). The vast majority of email work (such as recruiting network members to produce and review content) was done by LT and NM. The lack of engagement between meetings, and focus on discussion during meetings meant that engagement was limited to those present, and may have impacted on the group dynamics as well as motivation of those missing meetings.

The action group was empowered to act, and trusted by the steering committee, with a clear statement from members of the steering committee that the action group was free to decide on objectives and actions. This was further reinforced when proposed actions were taken up by the steering committee without any pushback or risk of decisions being reversed by others in the network. However, one action group participant fed back that they felt there needs to be a clearer understanding of the role of the action group versus that of the steering committee. This feedback has already been taken into account by the steering committee and will be implemented in the organisational structure, with a clearer division of roles between the steering committee (focusing on administrative and policy level) and the action group (focusing more on the network meeting content). This would further empower the action group through a better understanding of the roles of the action group and the steering committee. This may also impact on the make-up of the action group, where LT, for example, may not need to continue in their role in the action group.

### Roles and group dynamics

#### Researcher-in-residence

The RIR (NM) is a family doctor and researcher in PSS/FD with a background in leadership and management in non-governmental organisations. In this instance, the RIR primarily provided the roles of facilitator and driver of the process, researcher and research translator, negotiator for time and space within the network, facilitator of the process including action group and other meetings as well as being collaborator with network members and other partners. Often these roles, in particular, those of collaborator and negotiator were also held by LT, where connections were already present.

An important factor in this split of roles lies in qualities inherent to the RIR’s position. These included the transient nature of their role—their position was not permanent. Another important consideration is that the RIR is not from a Dutch cultural background, living in the country for less than 3 years, and with limited fluency in the language. These factors created barriers in communication, both in terms of language (requiring moments of needing clarification or even translation), and in terms of process and group facilitation. The RIR has a cultural background which is more hierarchical and less consensus-driven than Dutch culture as well as being less direct in communication than Dutch culture.^29^ These differences required significant adaptation to the communication style and meeting facilitation by NM to meet the preferences and needs of the action group. Feedback from action group members on the process suggests that adaptations were effective, with follow-up emails appreciated in providing a clear overview. The language barrier was not disruptive to some, with signs of understanding by the RIR through reviewing the main outcomes of discussion in English. Others did feel it interrupted the process somewhat.

A large part of the challenge inherent in the role of RIR within a PAR project lies in maintaining a balance. This is the balance between moving the project forward, while making sure that the project remains participatory. This meant ensuring that the views and ideas of those in the network and community are the driving force behind the actions taken. However, as has already been highlighted, at times (such as between meetings), action was limited and required more direction and action by the RIR. This was also seen in meetings where the action group seemed to expect more direct leadership from the RIR, stated directly by email from one action group member. This instance was one of many where the RIR had to reflect on their position in the group and how to handle decision-making and action-taking. Throughout, however, the main decision-making power was kept within the action group. This was maintained by the RIR asking open questions of the action group, and repeatedly stating that it is the action group’s views that matter in terms of what actions to take. At times, however, a selection of options was offered, based as much as possible on the ideas and concerns brought by the members of the action group, or from other network members.

A final consideration with the RIR lies in the importance given to sustainability. The RIR had time-limited involvement in the network and, therefore, the action group. For this reason, consideration was given to slowly making the RIR redundant throughout the process by handing over certain tasks when appropriate. This included chairing of sessions and access to data and other resources such as the network website administration. This had the added benefit of potentially making the action group members feel that their work was more likely to be of longer term impact, rather than stopped and forgotten once the window of the study ended.

#### Experts-by-experience

The involvement of experts-by-experience was intended from the initial plans for this study. Two of the experts-by-experience also had professional clinical experience, which allowed them to consider different perspectives from experience, and potentially made it easier for them to challenge other clinicians. Challenges, however, were encountered both at the beginning as well as throughout the study, which had an impact on group dynamics and the overall outcomes. First, it was considered important to have at least one expert-by-experience involved in each meeting from the first meeting, which was the case. We, therefore, recruited two experts-by-experience. A challenge that was not considered sufficiently in advance was that of having experts-by-experience being unable to participate due to financial limitations. Further to this, challenges still arose with regards to experts-by-experience having a sense of safety. This required paying more specific attention at times as well as phone calls to listen to the concerns and identify ways to improve the space during meetings to be safer and more equitable.

Reflecting on the process, it at times felt that the actions being taken were practitioner-focused, with concern being raised as to whether the involvement of experts-by-experience was sufficiently centred in our actions, or whether it was tokenistic. This challenge was aided by having vocal experts-by-experience, on more than one occasion drawing the attention of the action group to the challenges of experts-by-experience, including an interlude to read and reflect on a poem about living with illness (see osf.io/z8k6q). Throughout, actions were taken to ensure, for example, accessibility and centring of lived experience in the work. This included focusing the content produced for objective 2 on being accessible to potential patients, in particular, including ensuring the language is accessible with support of professionals who work in the field. We also decided to have a first video recorded by an expert-by-experience giving an overview from the patient’s perspective. Another decision made during the last meeting—after a suggestion from members of the group—was for one of the experts-by-experience to coauthor this paper. In this way, we could ensure that the work presented reflects the lived experience more authentically.

## Discussion

### Principal findings

With regards to our first question—the action processes undertaken by the action group to improve the CCN *ALK Netwerk Salland*: as a group of professionals providing PSS/FD care, this fell under three objectives. The first objective was to identify financial resources available to *ALK Netwerk Salland*, which were found to be strictly limited at the time, limiting the actions we could take. The second objective was to improve knowledge of treatment options through creation of content to explain what different disciplines do, how they can help people with PSS/FD and how to access the services of each discipline. This includes text content, which was specifically reviewed by a group of experts-by-experience to ensure the language is accessible and patient-centred, and made available online. The third objective was to improve the shared vision of care for PSS/FD through interactive activities at network meetings, to find a shared language, reach similar explanatory models and have feedback on how professionals communicate from other professionals from all kinds of disciplines and experts-by-experience.

With regard to the conditions for an effective change process, this study showed a number of important factors. It showed the importance of having a group of individuals working together, representing the different stakeholders from within the network, all with an understanding of the challenges posed by PSS/FD care. The importance of this group being empowered and trusted by the wider network is also recognised, as well as having a trusted, empowered and embedded RIR/lead facilitator. Such an RIR would be very important to facilitate the process and support the action group through research and team management. Part of the strength of a trusted and empowered RIR was also the link to a respected network member with knowledge of the network and its members. This connection assisted in being able to reach out to others in the network when needed, to improve the quality of the action group’s work. This is also aided by good team management both by the RIR and by others in the action group. The position of the RIR is also important in terms of the actuality and perception of them as being insiders or outsiders. In this case, the RIR was, in a way, both an outsider and an insider. Outsiders may have the advantages of bringing an outside perspective and a more objective perspective. Insiders have more trust from the rest of the team as well as insider knowledge.^30^ Therefore, in this case, the RIR was able to bring outsider knowledge, experience and perspective, alongside having the insider knowledge of, and connection with, respected network members.

However, as seen in the first objective, we also found that limited resources hampered our ability to effect change, due to lack of funds, which would allow for creation of higher quality content directly. The issue of lack of resources comes up against the recognition that often resources, especially financial, are only made available once a strategy is already proven effective. The lack of resources also limited the time that could be dedicated to the action group’s work, relying on the dedication and voluntary action of other action group members as well as on the RIR (initially) and network coordinator (latterly). The lack of resources, in particular financial, restricted not only how engaged members in the group could be but also who could be part of the action group at all. Challenges of financial stability, access to childcare, time off from clinical work and health all impacted on action group engagement and therefore the actions that could be taken. Such resources are also important to ensure meaningful involvement of experts-by-experience as one of the stakeholder groups, and therefore the centring of lived experience in the actions being taken. Time itself is an important resource—not only for enacting plans. It is also important to allow for the action group to get to know each other and build trust, and from there discuss different perspectives where there may be disagreement on what matters. This was the case in our action group, where several participants of the action group did not know each other prior to the group being formed, which may have been a factor in the slow process to build consensus.

### Strengths and limitations

The challenge of cultural and language barriers between the RIR and the rest of the action group impacted on the flow of meetings and therefore may have impacted on group dynamics. Measuring outcomes was often not easy or even possible. This, however, is a problem inherent to *ALK Netwerk Salland*, which is a loose network of professionals. Instead, we depended on the presence of a product as an outcome in itself rather than being able to measure the product’s impact. This was especially the case considering the time limitations of the study and the time available to action group members and other network members.

A number of strengths in this study are also noted. These included having research that reflects the experiences of the action group and network members, on which to base decisions and act. This was alongside the strengths of a diverse action group allowing for triangulation of ideas and action plans, bringing different perspectives and critique of possible plans. It is also important to note the involvement and work with experts-by-experience and other stakeholders in the community, which is an essential part of a participatory study.

An important strength inherent in the PAR method, and supported by a trusted and empowered RIR working closely with the action group, is the ability to use research methods and develop actions based on the needs and resources of the network in the current time. This allows for immediate evaluation of actions and reflection on next steps based on this evaluation. This is especially important in CCNs for PSS/FD, where evaluation has been a challenge.^10^

Finally, the in-built planning to keep the action group going once the study window ended meant that the work of the action group could continue, with the aim of increasing the impact of the actions taken. In this way, the process of this study primarily provided a framework around which to start working as well as giving the action group a sense that this is not about the research but about improving the quality of the CCN for PSS/FD.

### Comparison with the literature

Various examples of PAR in healthcare exist. These can vary, looking at improving a specific healthcare service,^31^ or can focus on improving healthcare culture;^32^ with variations in the involved participants as well. To our knowledge, no PAR studies exist looking at implementation of change in CCNs for PSS/FD care. Neither are there studies—using other methodologies—looking at the implementation process of CCNs in PSS/FD care or other areas in the field of psychosomatic medicine. In a recent systematic review looking at CCNs in PSS/FD,^10^ 39 CCNs are described. Of these, only two make reference to changes in the implemented interventions.^33 34^ However, no further description is given of the implementation of change process. Furthermore, due to the variation of what a PAR process can look like, it is difficult to compare our study with others using a similar PAR approach. Studies often focused on the personal experience of the process rather than the change^35^ or on the process of codesign of a product, without the consideration for implementation of the final product.^36^ What we do present in the form of our process, however, is a potential model for implementing change in an active network, being clear about the many obstacles, which will likely show how complicated it may be to achieve change (on objectives that may seem on face validity easy to achieve) in the real world and give recommendations on how to improve this process.

One of the challenges raised in this study is the process of consensus-building and decision-making. It is possible that this slow process may relate to uncertainty about the methodology being used, or unease in working in the action group as a result of uncertainty of the level of empowerment. This, however, does not fit the high engagement from the first meeting. Furthermore, the process we went through does fit experiences and expectations related to cultural differences.^29^ It is common in the Netherlands for consensus-building to be an important, and often slow, process. This is also reflected in the action group seeking the opinions of network members on what the action group should prioritise. The consensus-building process is normally followed by clear agreement on actions and therefore a reasonably quick process of achieving actions. This is in contrast to, for example, the UK, where decision-making is quicker, with action often taking longer as the decisions are questioned and may require changes due to the reduced consensus-building.^29^ The implications of this are that when planning a process of change, it is important to consider local context and culture. This is especially important when working across cultures or moving from one culture to another.

### Implications for practice and research

Creating a group of stakeholders to drive and implement change in a CCN for PSS/FD is clearly a realistic way to improve the service provided. This is especially the case within a PAR cycle, with inbuilt, real-time evaluation and reflection of actions, whether research is an important aim for the network in question or not. However, there are lessons to learn from our attempt.

First, an important recommendation to assist in any implementation of change process is to have a lead facilitator, implementation lead or RIR. There are different aspects to these roles, whether, depending on the needs of the PAR process and the local needs.^21^ This person would drive the overall change process as a process facilitator, facilitating and leading the implementation of change with stakeholders.^25^ It is important for the lead facilitator to be embedded within the network and to be empowered to facilitate the process, providing continuity and focus to the process. Careful consideration should be taken on whether this person should be an insider or an outsider and the experience and position they have. For example, a good understanding of the challenges related to PSS/FD care and an understanding of the health system in which this care is being provided seems crucial. More practical factors such as the time they can give to the change process, and their language and cultural competency in relation to the group they would be working with should also be as close as possible. This person should also take active interest in the group connection, the decision-making process and involvement of the whole action group.

Second, involving experts-by-experience and other stakeholders in true partnership as early in the process as possible, with a mandate from the organisation within which the PAR is taking place, increases the chances of the involvement being meaningful and impactful on the change process. This also increases the chances that the changes will be sustained. It is also strongly advisable for the involved experts-by-experience to be adequately compensated for their time, which relates to the need for funding to assist the change process. It is also important, in the spirit of embeddedness, that the priorities and actions of the action group appropriately represent the priorities of the network and those the network serves.

Third, with regards to assessing the impact of the changes implemented, serious consideration should be taken to measuring outcomes. Direct outcome measures may not be possible, requiring proxy measures. In some situations, subjective measures such as the questionnaire to assess perception of performance on CCN quality indicators, used at the *ALK Netwerk Salland* 6 monthly meeting (osf.io/pvza2), may be used. We would recommend building outcome measures with stakeholders embedded in the care process, in particular, experts-by-experience. Creating such outcome measures would likely require some innovative thinking as well as looking for inspiration from other sectors—strong engagement with all relevant stakeholders can aid in this process. The evaluation plan, as included in the objectives of the action group, is an essential part of the process. Website hits and service user surveys may be useful as planned in the action group objectives. Other options may be realist evaluation, as being undertaken in *ALK Netwerk Salland* in follow-up research, where quantitative and qualitative data as well as log data are combined (osf.io/8jzny).

### Conclusion

It is possible with intrinsically motivated people to implement change in the CCN for PSS/FD using a PAR approach. Action processes that can have an impact include creating accessible information of the treatment options, and moving towards a shared vision of care by creating spaces for a shared understanding of PSS/FD to develop. However, to implement change more effectively and sustainably, the action group needs a clear mandate, financial resources and preferably a trusted RIR/lead facilitator, with active inclusion of experts-by-experience. A rigorous process for assessing outcomes and evaluating is also an important element to ensure effective implementation of change.

## Supplementary material

10.1136/bmjopen-2025-107978 online supplemental file 1

## Data Availability

Data are available in a public, open access repository. Data are available upon reasonable request.
