# Supplementary material for: Improving collaborative care networks for functional disorders and persistent somatic symptoms: a participatory action research study in the Netherlands
Source: BMJ Open. 2025 Dec 12;15(12):e107978. doi: 10.1136/bmjopen-2025-107978 (PMC12706206; doi:10.1136/bmjopen-2025-107978)
Supplement: online supplemental file 1 [file bmjopen-15-12-s001.docx]

**Metadata**

**Title**

Improving collaborative care networks for functional disorders: A Participatory Action Research study

**Description**

There is a widespread belief that collaborative care networks (CCN) have the potential to significantly improve outcomes in the care of functional disorders (FD) and persistent somatic symptoms (PSS). However, evidence of the effectiveness of CCNs is lacking. The first steps are being taken - defining CCNs, outlining the characteristics that have been considered, as well as identifying quality indicators. The next step is applying what has been learnt into practice. We argue that the best way to do this is with an active network, and with the involvement of the network members themselves as this not only has the possibility of directly improving a service, it also gives a more realistic view of what works, as well as giving an immediate process of trial and error. Therefore, in this study we will use participatory action research (PAR) to incorporate and enact the learning we have made bringing this together with the experience of an active network and its members. In this case, we will be working with ALK Netwerk Salland, a network of healthcare professionals across the Salland region in the east of the Netherlands, who work with persons suffering with FD and PSS (*in dutch: aanhoudende lichaamlijke klachten - ALK*).

**Contributors**

[Nick Mamo](https://osf.io/d7eqs)

Judith Rosmalen

Lineke Tak

Denise Hanssen

Tim olde Hartman

ETUDE

**Category**

 Project

**Affiliated institutions**

*Dimence*

*UMCG*

*RadboudUMC*

**License**

CC-By Attribution 4.0 International

**Subjects**

- Social and Behavioral Sciences
- Medicine and Health Sciences
- Organization Development

**Tags**

*No tags*

**Study Information**

Research Aims

The aim of this study is to see what can improve the work and outcomes of ‘ALK Netwerk Salland’. This will be done through a participatory cyclical process of improving the same network by identifying objectives and related actions, and associated evaluation. This is with the ultimate aim of improving outcomes of persons living with FD and PSS, both directly by improving this network, and by providing realistic ways to improve other networks.

This can be done through a number of possible routes such as improving the communication processes within the network and improving the coherence and agreement in the language used by all network members with regards to explanation of conditions and treatment aims. As this will use a participatory process the detailed objectives and related actions will be defined by the research committee that will be set up as part of the process.

If helpful, please select the type of aim (non-exhaustive list):

Describing

(other options including exploring, theory evaluating, comparing, understanding).

Research question(s)

*What action processes can improve the quality of a CCN for FD?*

Anticipated Duration

Start: 05/2023

End: 05/2024

**Design Plan**

Study design

This study will utilise Participatory Action Research (PAR) methodology. This will mean that other research methods will be employed depending on the objectives and actions decided on by the research committee. The core research process of PAR is a cycle of:

1. Problem definition - including data collection and analysis, which also takes the form of evaluation on further cycles
2. Reflection - on the outcomes of the data and evaluation
3. Planning - identification of actions targeting to the data outcomes
4. Action - implementing planned actions

Sampling and case selection strategy

The study will take place within ALK Netwerk Salland, which is made up of healthcare professionals providing care for persons with FD and PSS. The Research Committee will be made up of members of the network, each representing one or more of the six levels of healthcare as described by Grol and Wensing (https://doi-org.proxy-ub.rug.nl/10.5694/j.1326-5377.2004.tb05948.x). The six levels are innovation, individual professional, patient, social context, organisational context, and economic and political context. A minimum of one person representing each level is essential (though one person may represent two levels), and two persons representing the patient/experience expert level. The persons involved must be willing to commit to regular meetings for the duration of the study. The role of the Research Committee is to decide on objectives and actions to improve the active network using data already available, as well as with the option of collecting and analysing new data. The role of the main researcher within this committee is to facilitate meetings, and to provide support by providing relevant resources (including literature). The reason behind this strategy is to ensure that the research is participatory (Freire, 1970 - Pedagogy of the oppressed) and embedded in the local context, as well as being reflexive and transparent.

**Data Collection**

Data source(s) and data type(s)

Data available before the study:
- Delphi study into quality indicators for CCN for FD by lead author (https://osf.io/f9d5x)
- Systematic review on characteristics of CCN for FD by lead author (https://osf.io/4gv2t)
- Nominal group techinque study by lead author (https://osf.io/wg49u/)
- Other relevant studies (including but not limited to Delphi study and Systematic review into barriers and facilitators to implementation of services for medically unexplained symptoms (MUS) by Dr D. Hanssen)

Data in process:
- Unpublished survey data into implementation of services for and attitudes torwards MUS by Dr D. Hanssen
- Evaluation questionnaire designed using the top fifteen indicators identified in the lead author's Delphi study
- Outcomes from workshops identifying ways to apply the top fifteen indicators identified in the lead author's Delphi study (at time of writing, two workshops have been undertaken on this subject - in September and November 2022 with healthcare professionals involved in FD and PSS care across the Netherlands, and consultants working in care networks across the Netherlands, respectively).

Data collection methods

Data collection methods beyond those already described above will be defined based on the objectives defined by the research committee.

The evaluation questionnaire described above will be undertaken on a cyclical basis and will provide a basis for evaluating as well as deciding on areas for improvement. This questionnaire utilises a Likert scale, asking respondents to evaluate how they think the network is currently performing in a number of areas.

Data collection tools, instruments or plans

- Evaluation questionnaire as shown - Other tools to be defined by research committee depending on defined objectives

- [Vragenlijst over de kwaliteit van de netwerken copy.docx](https://osf.io/bktnf/files/osfstorage/63f370eeb680bc01f84e421a)

Stopping criteria

Time limitation - intial phase of this study must be completed before 31/08/2024 due to contract and funding limitations.

**Analysis Plan**

Data analysis approach

Primary data analysis will be based on an objectives template form which will bring together the objectives and their associated action plans, evaluation plans and outcomes. These will be defined by the PAR committee, however the documentation and overall outcomes of these will be presented in table form.

Further analysis will depend on the objectives and their associated action plans, evaluation plans and outcomes as defined by the PAR committee during the process of the study.

Data analysis process

Analysis will depend on on the objectives and their associated action plans, evaluation plans and outcomes as defined by the PAR committee during the process of the study. The PAR committee will be the primary team involved in defining the data to be collected, the methods of analysis and the actions to be taken in response. Additional support will be from the other authors in the study.

Credibility strategies

- Member checking
- Bringing in different perspectives
- Consensus building among team members or 'interrater reliability'
- Reflexitivity
- Dialogues with subjects
- Other (please explain)

Please provide a short rationale for why you selected particular strategies and how they are appropriate given your study’s aim(s) and approach, or specify your credibility strategies if not on the above list.

As previously described, PAR is based on core principles of participation and embeddedness (therefore involving members of the studied community within the local context), reflexivity (especially with regards to the participatory process and positionality of the researcher), as well as the importance of both academic development and social change, and transparency. For this reason, member checking, bringing in different perspectives, consensus building among team members, reflexivity and dialogues with subjects are essential. Depending on the methods selected, other strategies will be utilised.

**Miscellaneous**

Reflection on your positionality (optional)

Within PAR this is an essential point to consider. The positionality of the lead author is integral to the PAR process. Important factors to consider within this are hierarchy, relationship to the studied population, and role within the PAR committee. With respect to these, it is important for there not to be a sense of significantly different hierarchical positions within the research committee, especially not in the case of the lead author setting up the research committee. This also relates to the relationship to the studied population. There is benefit of a certain distance allowing for a more objective view of the work being done, however it is also important for the studied population to trust the lead author in what they can and will provide to the research project. Therefore, relevant credentials must be considered. In this case, being a reliable healthcare professional with some relevant experience is of use here, along with already having research data with which the studied population can more easily identify. Finally, it is important with all this in mind, to make clear the role of the lead author within the PAR committee. Specifically, the lead author must make clear that they are not in a position of power over the rest of the committee, instead being in a position to facilitate the committee, both during the meetings (by providing an agenda and utilising facilitation techniques to support the flow of the meetings) as well as by providing other resources during and between meetings (including but not limited to relevant literature).
