# Supplementary material for: Improving collaborative care networks for functional disorders and persistent somatic symptoms: a participatory action research study in the Netherlands
Source: BMJ Open. 2025 Dec 12;15(12):e107978. doi: 10.1136/bmjopen-2025-107978 (PMC12706206; doi:10.1136/bmjopen-2025-107978)
Supplement: Supplementary file 2 [file bmjopen-2025-107978.reviewer_comments.pdf]

PEER REVIEW HISTORY

BMJ Open publishes all reviews undertaken for accepted manuscripts. Reviewers are asked to complete a checklist review form and are provided with free text boxes to elaborate on their assessment. These free text comments are reproduced below.

ARTICLE DETAILS

Title (Provisional)

Improving collaborative care networks for functional disorders and persistent somatic symptoms: A Participatory Action Research study in The Netherlands

Authors

Mamo, Nick; Hanssen, Denise J.C.; Korten, Sylvia; Olde Hartman, Tim C.; Rosmalen, Judith GM; Tak, Lineke

---

VERSION 1 - REVIEW

**Reviewer**	**1**
**Name**	**Guthrie, Elspeth**
**Affiliation**	**University of Leeds Leeds Institute of Health Sciences, Psychological and Social Medicine**
**Date**	**12-Aug-2025**
**COI**	**None**

This paper is very well written and clear. It describes the process of using participatory action (PAR) research to implement change in a collaborative care network for people with functional and/or persistent symptoms. My area of expertise is persistent physical symptoms rather than PAR. So I am not that familiar with the specific research methodology. The authors meticulously describe the process of trying to implement 3 objectives to improve a collaborative care network in the Netherlands. Although, the health system in this country, is different to others, the barriers to implementing change which the authors describe are fairly universal. I think the paper captures the enormous difficulties involved in implementing change in health systems, even by well intentioned and highly motivated individuals. The paper is really a descriptive account of that process of change but this kind of research approach is relatively new in this setting and will be of interest to readers of the journal. Not being familiar with PAR, it would be helpful if the authors could provide perhaps an example in another field of medicine where it has been used to good effect. Also, there seem to be overlaps with quality improvement programmes (QIP) which are fairly common in health service settings, so some explanation of the contrast between these approaches and PAR would be helpful.

**Reviewer**	**2**
**Name**	**O'Neal, Mary**
**Affiliation**	**Brigham and Women's Hospital, Department of Neurology**
**Date**	**07-Oct-2025**
**COI**	**None**

Thank you for writing a clear pathway to implement change in a CCN for patients with PSS and FND. I especially thought the section around group dynamics was helpful to consider when using experts by experience and why careful thought as to the RIR is critical. It would be helpful to consider innovative ways you could measure outcomes in next steps- number of hits on the website, patient surveys for example.

I do have a couple of minor suggestions:

PSS is better defined as physical symptoms that last for an extended period and are distressing or disruptive to daily life, regardless of whether there is a medical explanation.

Please define how functional neurologic disorder and PSS are different. The approved name is FND and that should be used in the manuscript.

In the design section please indicate after participatory action research initially that you will be referring to that as PAR or don't use the initials in the strengths and weakness section

VERSION 1 - AUTHOR RESPONSE

Reviewer: 1

> *Dr. Elspeth Guthrie, University of Leeds Leeds Institute of Health Sciences*
> *Comments to the Author:*
> *This paper is very well written and clear. It describes the process of using participatory action (PAR) research to implement change in a collaborative care network for people with functional and/or persistent symptoms. My area of expertise is persistent physical symptoms rather than PAR. So I am not that familiar with the specific research methodology. The authors meticulously describe the process of trying to implement 3 objectives to improve a collaborative care network in the Netherlands. Although, the health system in this country, is different to others, the barriers to implementing change which the authors describe are fairly universal. I think the paper captures the enormous difficulties involved in implementing change in health systems, even by well intentioned and highly motivated individuals. The paper is really a descriptive account of that process of change but this kind of research approach is relatively new in this setting and will be of interest to readers of the journal. Not being familiar with PAR, it would be helpful if the authors could provide perhaps an example in another field of medicine where it has been used to good effect.*

To highlight examples of good PAR in healthcare research, the following has been added to the comparison to literature section:

Various examples of PAR in healthcare exist. These can vary, looking at improving a specific healthcare service [30], or can focus on improving healthcare culture [31]; with variations in the involved participants as well.

Also, there seem to be overlaps with quality improvement programmes (QIP) which are fairly common in health service settings, so some explanation of the contrast between these approaches and PAR would be helpful.

Indeed, PAR is strongly linked to quality improvement projects, in particular the PDSA cycle. The following note has been added to the section on study design to highlight this, as well as the key difference:

This study design differs from quality improvement projects, in particular those using the PDSA (plan-do-study-act) cycle often seen in healthcare. The primary difference lies in PAR's focus on active participation of key stakeholders who form part of the healthcare service. In particular, this includes the 'service users '- persons with PSS/FD who have needed or currently need the healthcare service.

Reviewer: 2
Dr. Mary O'Neal, Brigham and Women's Hospital
Comments to the Author:
Thank you for writing a clear pathway to implement change in a CCN for patients with PSS and FND. I especially thought the section around group dynamics was helpful to consider when using experts by experience and why careful thought as to the RIR is critical. It would be helpful to consider innovative ways you could measure outcomes in next steps- number of hits on the website, patient surveys for example.

Thank you for this point. I have added the following to the 'Implications for practice and research', expanding on the assessment of impact:

Creating such outcomes measures would likely require some innovative thinking as well as looking for inspiration from other sectors – strong engagement with all relevant stakeholders can aid in this process. The evaluation plan, as included in the objectives of the action group, is an essential part of the process. Website hits and service user surveys may be useful as planned in the action group objectives. Other options may be realist evaluation, as being undertaken in *ALK Netwerk Salland* in follow-up research, where quantitative and qualitative data as well as log data are combined (osf.io/8jzny).

I do have a couple of minor suggestions:
PSS is better defined as physical symptoms that last for an extended period and are distressing or disruptive to daily life, regardless of whether there is a medical explanation.

Thank you for highlighting this - I have added in the introduction the clarification that the physical symptoms are distressing.

Please define how functional neurologic disorder and PSS are different. The approved name is FND and that should be used in the manuscript.

The following explanation has been added to the introduction:

Often also referred to as functional somatic disorders (FSD), FDs include multiple disorders such as functional neurological disorder (FND) and irritable bowel syndrome, among others. Such disorders are typified by recognised clusters of PSS.

This clarifies both the relationship between PSS and FD, and between FD and FND. In this instance, we are taking FD to be an umbrella term that includes a number of disorders, including but not limited to FND.

> *In the design section please indicate after participatory action research initially that you will be referring to that as PAR or don't use the initials in the strengths and weakness section*

That you for noting my error in the strengths and weaknesses section. I have expanded the abbreviation in that instance.
